# Supplementary material for: Photodynamic Effectiveness of Copper-Iminopyridine Photosensitizers Coupled to Zinc Oxide Nanoparticles Against Klebsiella pneumoniae and the Bacterial Response to Oxidative Stress
Source: Int J Mol Sci. 2025 Apr 28;26(9):4178. doi: 10.3390/ijms26094178 (PMC12071902; doi:10.3390/ijms26094178)
Supplement: Supplementary file 1 [file ijms-26-04178-s001.zip › ijms-3518249-supplementary.pdf]

General synthesis and structural characterization of Cu(I) complexes

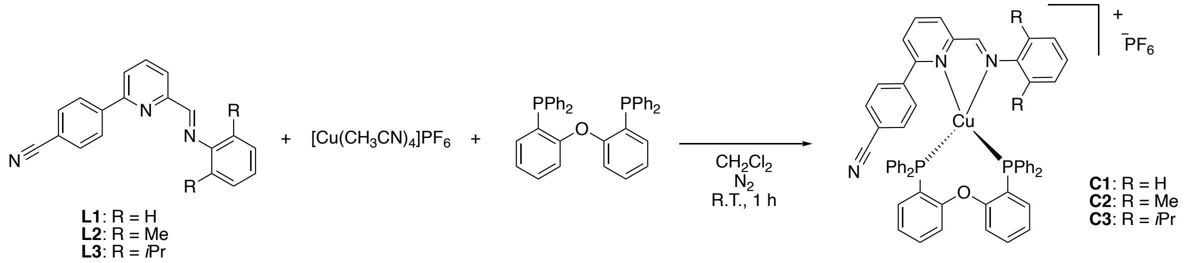

In a glass vial, one equiv. of $[Cu(CH_3CN)_4]BF_4$, one equiv. of DPEphos and one equiv. of the corresponding **L1-3** were added.[1] Then, the vial was sealed with a septum and was nitrogen flushed for 5 min. Later, 3 mL of anhydrous dichloromethane was added through a purged syringe and the reaction mixture was stirred at room temperature for 1 hour, under a nitrogen atmosphere. Afterwards, the volatiles were removed under reduced pressure and the crude product was purified by crystallization from a mixture of CH_2Cl_2 and toluene at -20 °C.

[(4-(6-((phenylimino)methyl)pyridin-2-yl)benzonitrile)(oxybis(2,1-phenylene))bis(di-phenylphosphane)Cu(I)]PF_6 (**CuC1**)

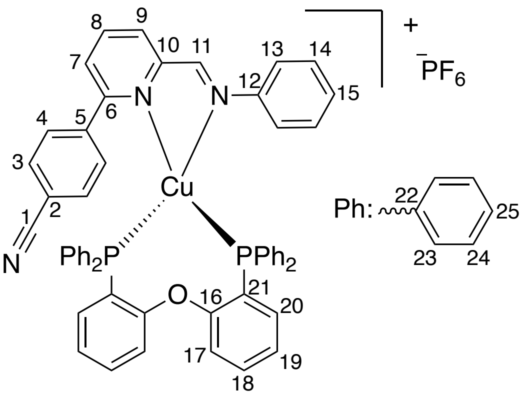

^{1}H NMR (400 MHz, $CDCl_3$, 298 K): δ/ppm 8.69 (s, 1H, H11), 8.23 (d, J = 4.4 Hz, 2H, H7, H9), 7.67 (t, J = 4.6 Hz, 1H, H8), 7.36 (dt, J = 16.5, 7.5 Hz, 4H, H25), 7.25 – 7.16 (m, 10H, H24, H3), 7.09 (t, J = 7.4 Hz, 1H, H15), 7.02 (d, J = 8.1 Hz, 2H, H4), 6.97 – 6.89 (m, J = 2.4 Hz, 10H, H23, H14), 6.76 (broad, 4H, H20, H19), 6.69 (d, J = 7.9 Hz, 2H, H18), 6.57 (m, 2H, H17), 6.49 (d, J = 7.8 Hz, 2H, H13).

$^{13}C\{^{1}H\}$ NMR (101 MHz, $CDCl_3$, 298 K): δ/ppm 162.4 (C11), 158.8 (C6), 156.8 (t, $J^{C\text{-}P}$= 5.7 Hz, C16), 151.1 (C10), 148.8 (C12), 143.2 (C5), 139.9 (C7), 133.8 (C17), 133.2 (dt, $J^{C\text{-}P}$= 11.7, 7.7 Hz, C20), 132.2 (C4), 130.7 (d, $J^{C\text{-}P}$= 7.1 Hz, C25), 130.6 (m, C24), 129.8 (t, $J^{C\text{-}P}$ = 16.6 Hz, C21), 129.2 (C9), 128.8 (C8, C23, C19), 128.6 (C3), 127.4 (C15), 124.8 (C14), 122.9 (t, $J^{C\text{-}P}$= 15 Hz, C22), 121.4 (C13), 119.6 (C18), 118.1 (C2), 112.8 (C1).

^{19}F NMR (400 MHz, $CDCl_3$, 298 K): δ/ppm -73.00 (d, $J^{F\text{-}P}$ = 712 Hz, PF_6).

$^{31}P\{^{1}H\}$ NMR (160 MHz, $CDCl_3$, 298 K): δ/ppm -13.82 (s, POP), -144.21 (hept, $J^{P\text{-}F}$ = 712 Hz, PF_6).

IR (KBr): ν/cm^{-1} 2230 (C≡N), 1589 (C=N), 1222 (O-C), 841 (PF_6).

Elemental analysis ($C_{55}H_{41}CuN_3OP_2$): calc: C 64.11; H 4.01; N 4.08; O 1.55. Found: C 65.94; H 4.15; N 4.02; O 1.62.

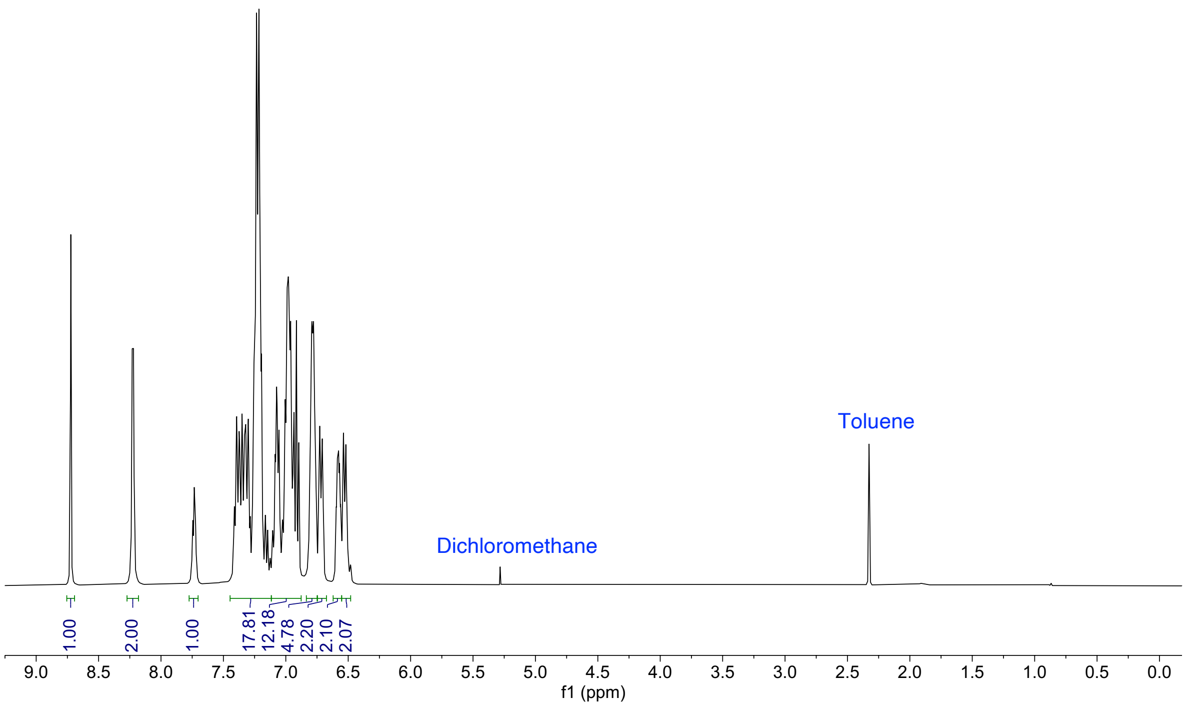

Figure S1. ^{1}H NMR (400 MHz, $CDCl_3$, 298 K).

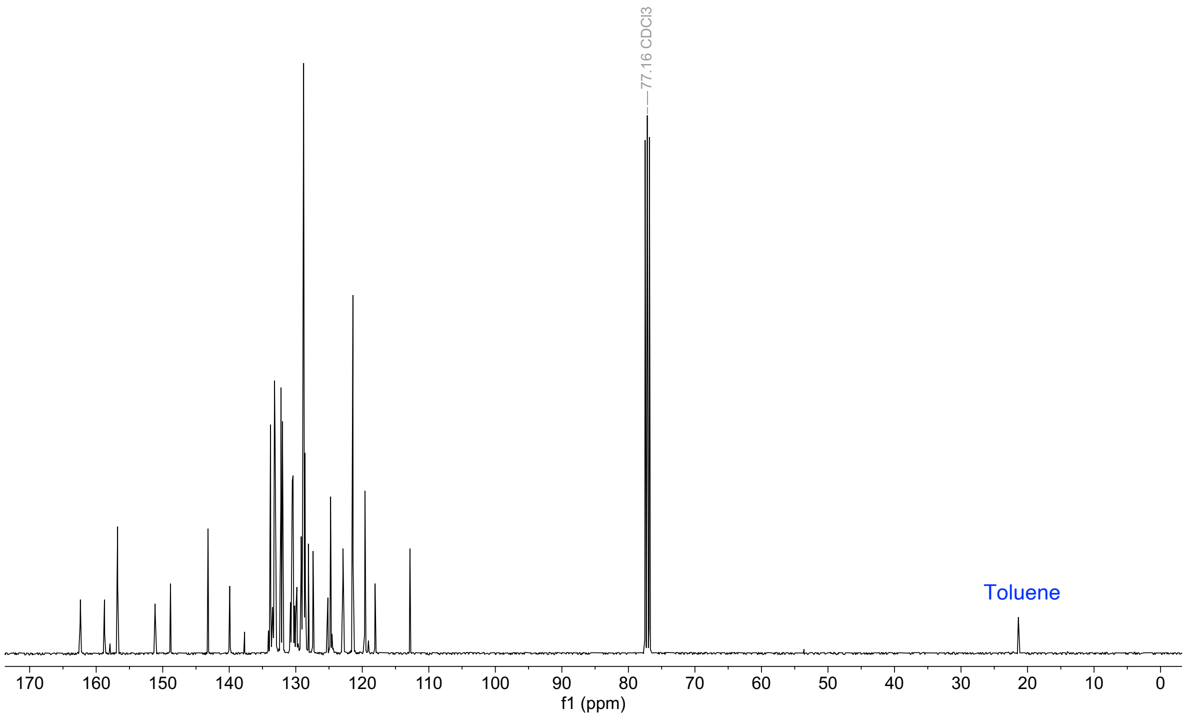

Figure S2. $^{13}C\{^{1}H\}$ NMR (100 MHz, $CDCl_3$, 298 K)

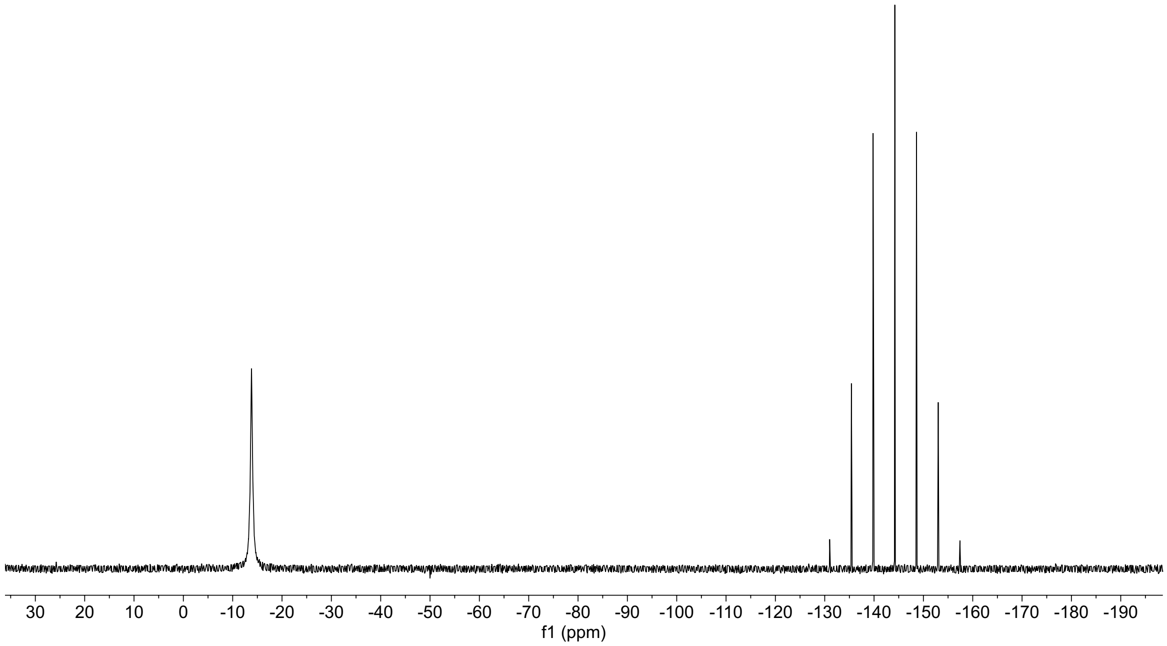

Figure S3. ^{31}P NMR (160 MHz, $CDCl_3$, 298 K)

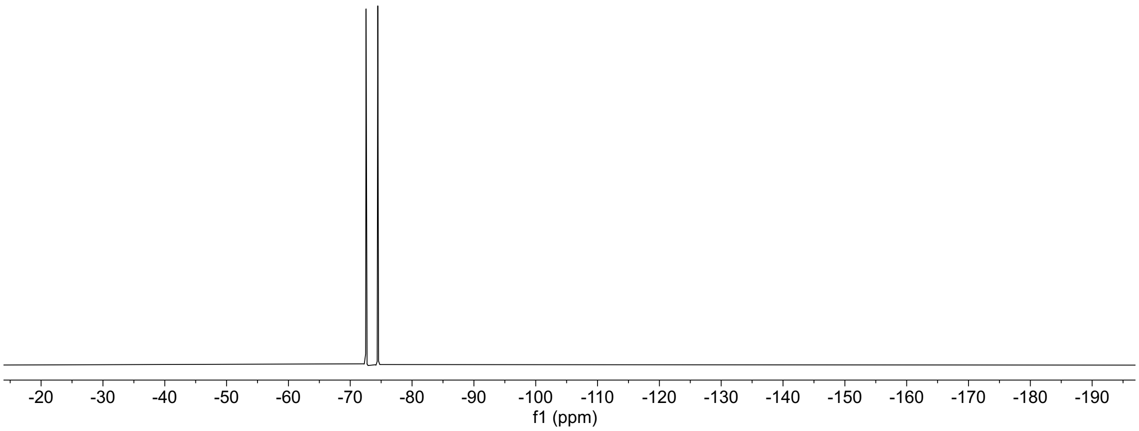

Figure S4. ^{19}F NMR (400 MHz, $CDCl_3$, 298 K)

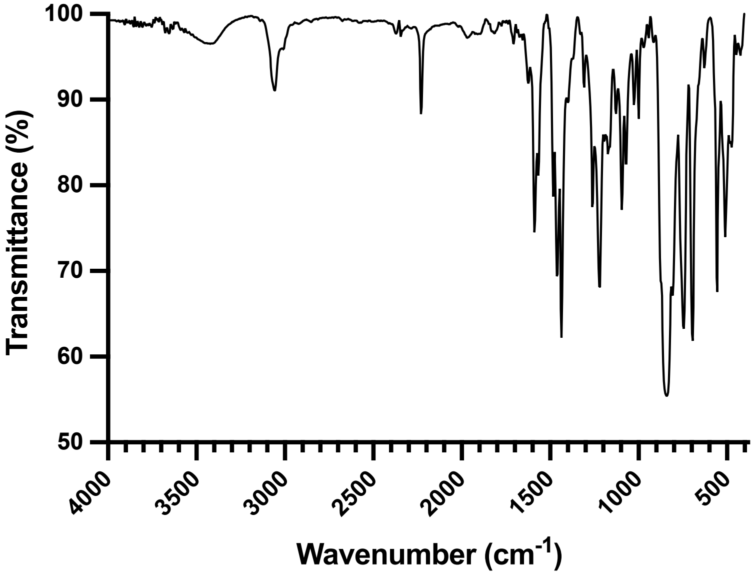

Figure S5. FT-IR spectrum of **CuC1** in KBr pellet.

[(4-(6-(((2,6-dimethylphenyl)imino)methyl)pyridin-2-yl)benzonitrile)(oxybis(2,1-phenylene))bis(di-phenylphosphane)Cu(I)]PF_6 (**CuC2**)

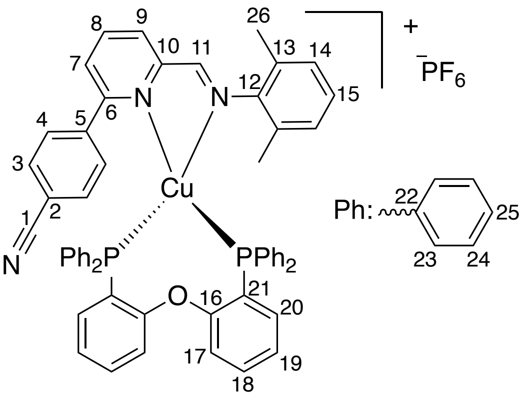

^{1}H NMR (400 MHz, $CDCl_3$, 298 K): δ/ppm 8.36 (m, 1H, H8), 8.34 (s, 1H, H11), 8.01 (d, *J* = 7.6 Hz, 1H, H9), 7.93 (d, *J* = 7.9 Hz, 1H, H7), 7.43 – 7.31 (m, 6H, H3, H25), 7.25 (broad, 1H, H25), 7.19 – 6.95 (m, 3H, H15, H19), 6.85 (broad, 1H, C14), 6.76 (broad, 10H, H20, H24), 6.64 (d, *J* = 7.9 Hz, 2H, H4), 6.44 (broad, 4H, H17, H18), 1.56 (s, 6H, H26).

$^{13}C\{^1H\}$ NMR (101 MHz, $CDCl_3$, 298 K): δ/ppm 166.4 (C11), 159.0 (C6), 156.5 (broad, C16), 150.6 (C10), 148.7 (C12), 142.6 (C5), 140.3 (C8), 134.1 (d, $J^{C\text{-}P}$ = 6.2 Hz, C17), 133.6 (broad, C20), 132.5 (C4), 132.3 (broad, C24), 130.5 (m, C25), 130.2 (C7), 129.7 (m, C21), 129.6 (C9), 129.2 (broad, C23), 129.0 (C14), 128.6 (broad, C19), 128.5 (C3), 128.1 (C13), 126.2 (C15), 122.8 (t, $J^{C\text{-}P}$ = 14.7 Hz, C22), 119.4 (C2), 119.1 (C18), 111.4 (C1).

^{19}F NMR (400 MHz, $CDCl_3$, 298 K): δ/ppm -73.69 (d, $J^{F\text{-}P}$ = 713 Hz, PF_6).

$^{31}P\{^1H\}$ NMR (160 MHz, $CDCl_3$, 298 K): δ/ppm -14.58 (s, POP), -144.29 (hept, $J^{P\text{-}F}$ = 713 Hz, PF_6).

IR (KBr): ν/cm^{-1} 2230 (C≡N), 1589 (C=N), 1220 (O-C), 841 (PF_6).

Elemental analysis ($C_{57}H_{45}CuF_6N_3OP_3$): calc: C 64.68; H 4.29; N 3.97; O 1.51. Found: C 62.62; H 4.49; N 3.83; O 1.58.

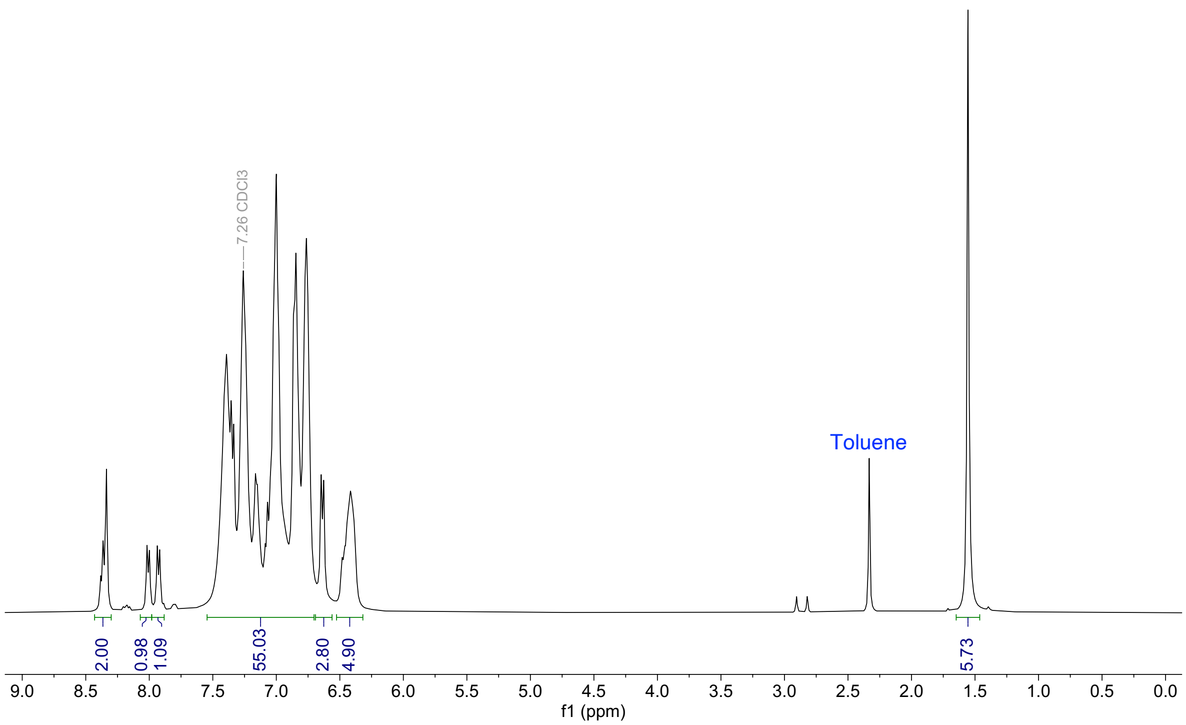

Figure S6. ^{1}H NMR (400 MHz, $CDCl_3$, 298 K).

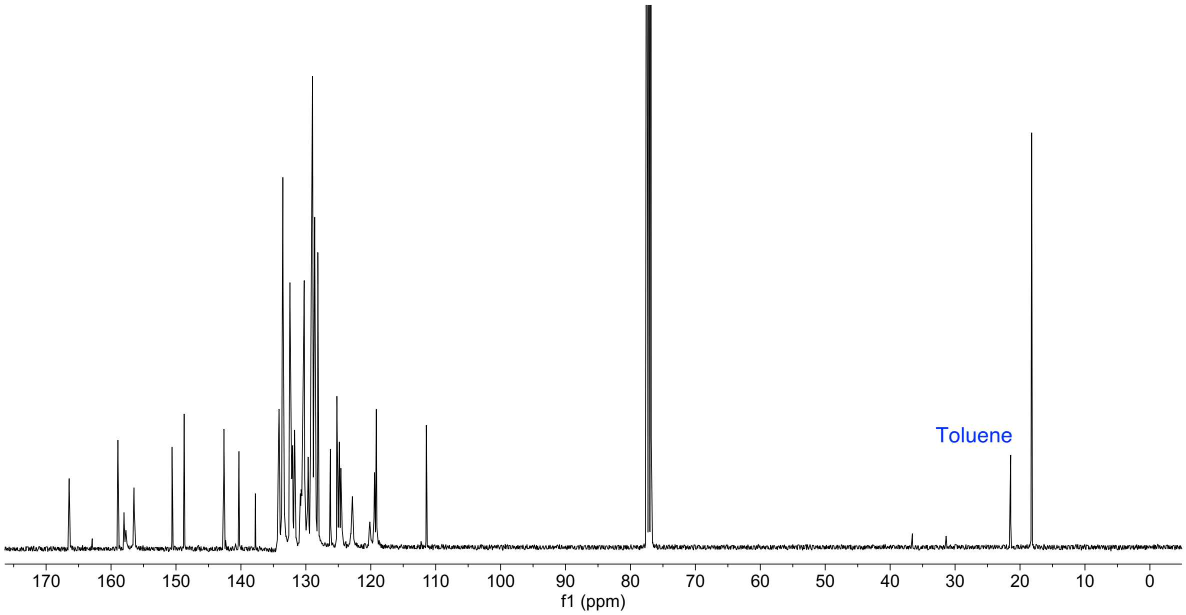

Figure S7. $^{13}C\{^{1}H\}$ NMR (100 MHz, $CDCl_3$, 298 K)

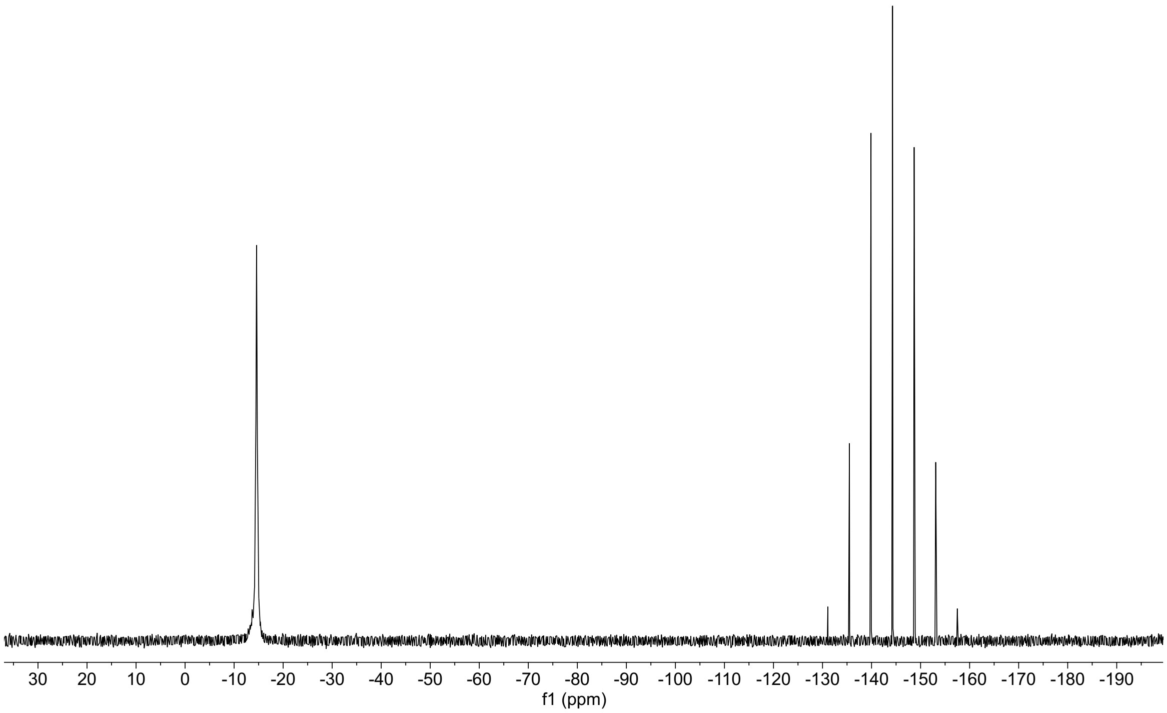

Figure S8. ^{31}P NMR (160 MHz, $CDCl_3$, 298 K)

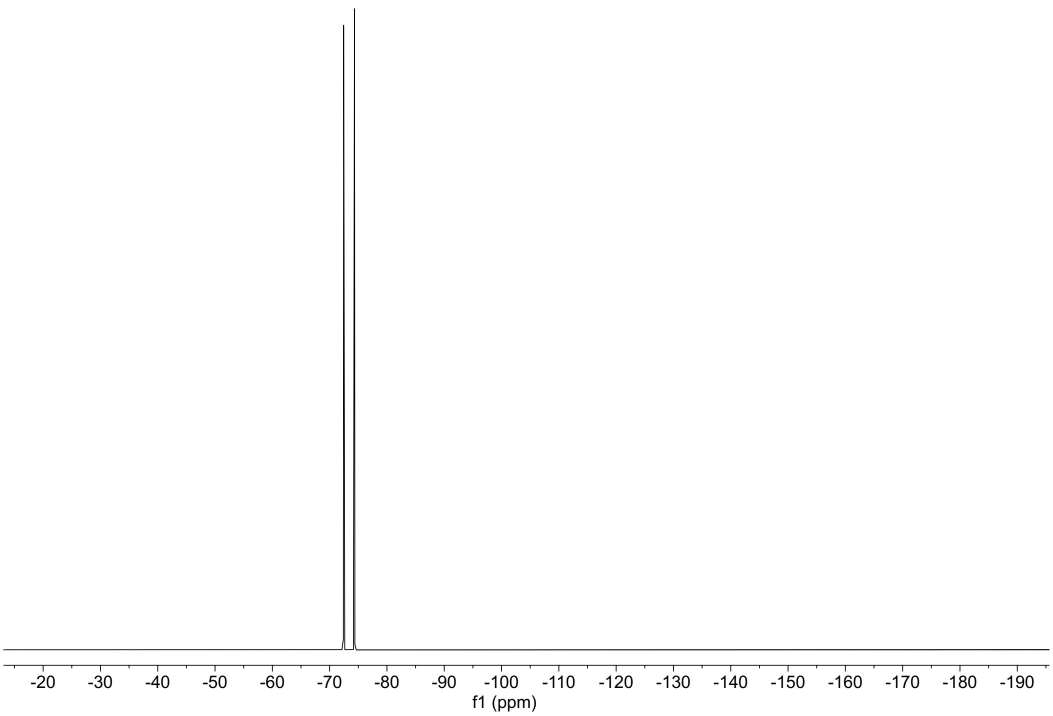

Figure S9. ^{19}F NMR (400 MHz, $CDCl_3$, 298 K)

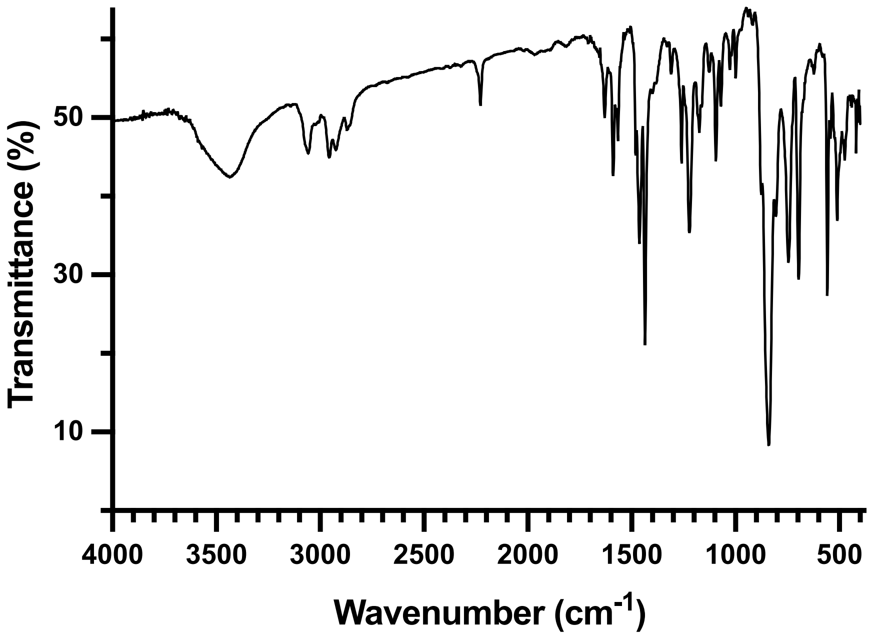

Figure S10. FT-IR spectrum of **CuC2** in KBr pellet.

[(4-(6-(((2,6-diisopropylphenyl)imino)methyl)pyridin-2-yl)benzonitrile)(oxybis(2,1-phenylene))bis(di-phenylphosphane)Cu(I)]PF_6 (**CuC3**)

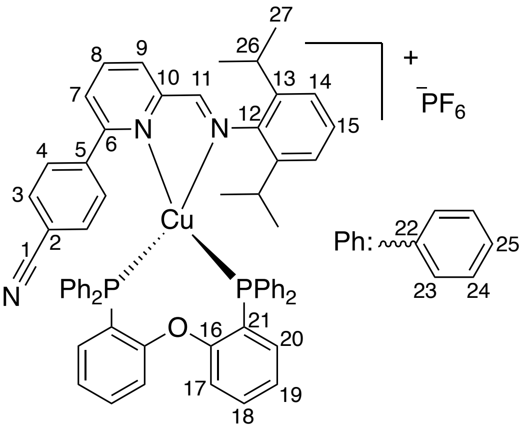

^{1}H NMR (400 MHz, $CDCl_3$, 298 K): δ/ppm 8.48 (t, J = 7.8 Hz, 1H, H8), 8.33 (s, 1H, H11), 8.03 (d, J = 8.0 Hz, 1H, H7), 7.94 (d, J = 7.6 Hz, 1H, H9), 7.50 – 7.43 (m, 2H, H3), 7.35 (broad, 9H, H15, H23), 7.17 (broad, 4H, H25), 7.09 – 6.99 (m, 4H, H4, H20), 6.89 – 6.83 (m, 10H, H19, H24), 6.71 – 6.62 (m, 2H, H14), 6.41 – 6.36 (m, 4H, H17, H18), 2.74 – 2.64 (m, 2H, H26), 0.63 (dd, J = 28.5, 6.7 Hz, 12H, H27).

^{13}C{^{1}H} NMR (101 MHz, $CDCl_3$, 298 K): δ/ppm 166.3 (C11), 159.0 (C6), 156.6 (broad, C16), 150.4 (C10), 147.2 (C12), 141.6 (C5), 140.8 (C8), 139.0 (C13), 134.15 (t, J^{C-P} = 8.3 Hz, C24), 133.8 (broad, C17), 132.7 (C4), 131.9 (C14), 130.7 (broad, C21), 130.6 (broad, C25), 130.4 (C7), 129.7 (C9), 129.5 (broad, C23), 128.3 (C3), 127.4 (C15), 124.9 (C19), 124.0 (C20), 123.2 (broad, C22), 119.7 (broad, C18), 118.7 (C2), 112.3 (C1), 28.3 (C26), 25.8 (C27b), 21.5 (C27a).

^{19}F NMR (400 MHz, $CDCl_3$, 298 K): δ/ppm -73.22 (d, J^{F-P} = 713 Hz, PF_6).

^{31}P{^{1}H} NMR (160 MHz, $CDCl_3$, 298 K): δ/ppm -14.78 (s, POP), -144.25 (hept, J^{P-F} = 713 Hz, PF_6).

IR (KBr): ν/cm^{-1} 2227 (C≡N), 1589 (C=N), 1221 (O-C), 841 (PF_6).

Elemental analysis ($C_{61}H_{53}CuF_6N_3OP_3$): calc: C 65.74; H 4.79; N 3.77; O 1.44. Found: C 68.52; H 4.93; N 3.64; O 1.41.

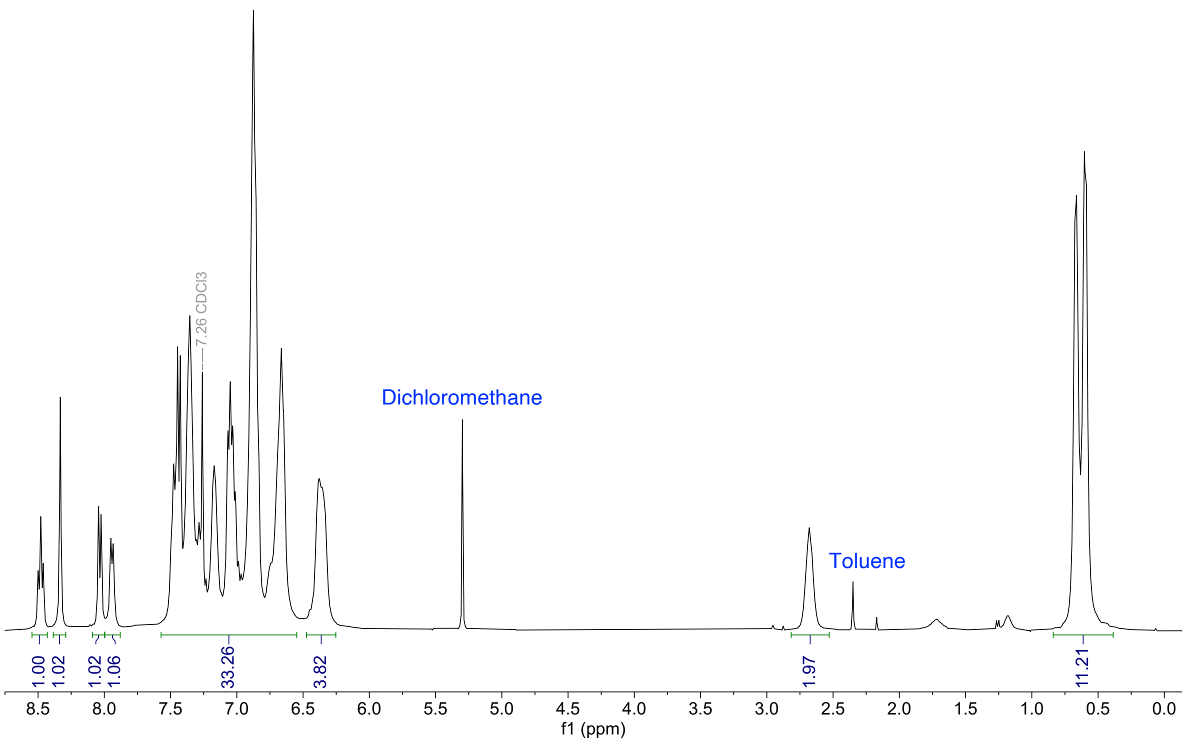

Figure S11. ^{1}H NMR (400 MHz, $CDCl_3$, 298 K).

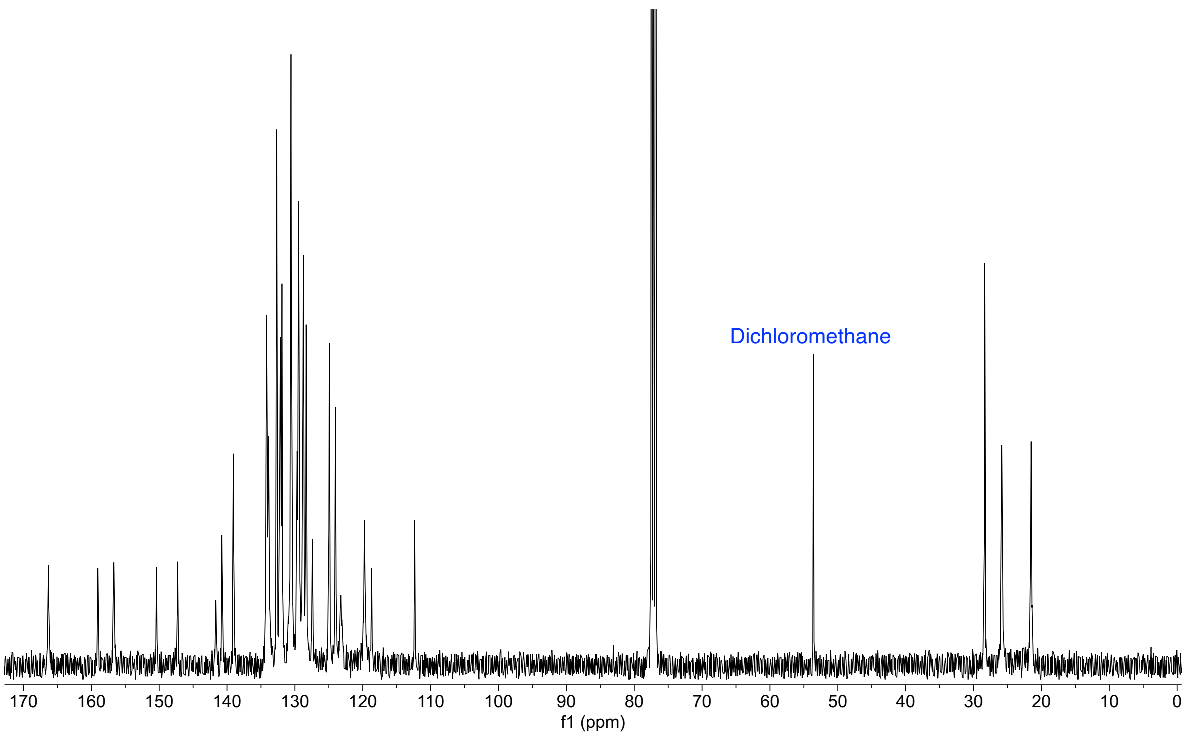

Figure S12. $^{13}C\{^{1}H\}$ NMR (100 MHz, $CDCl_3$, 298 K).

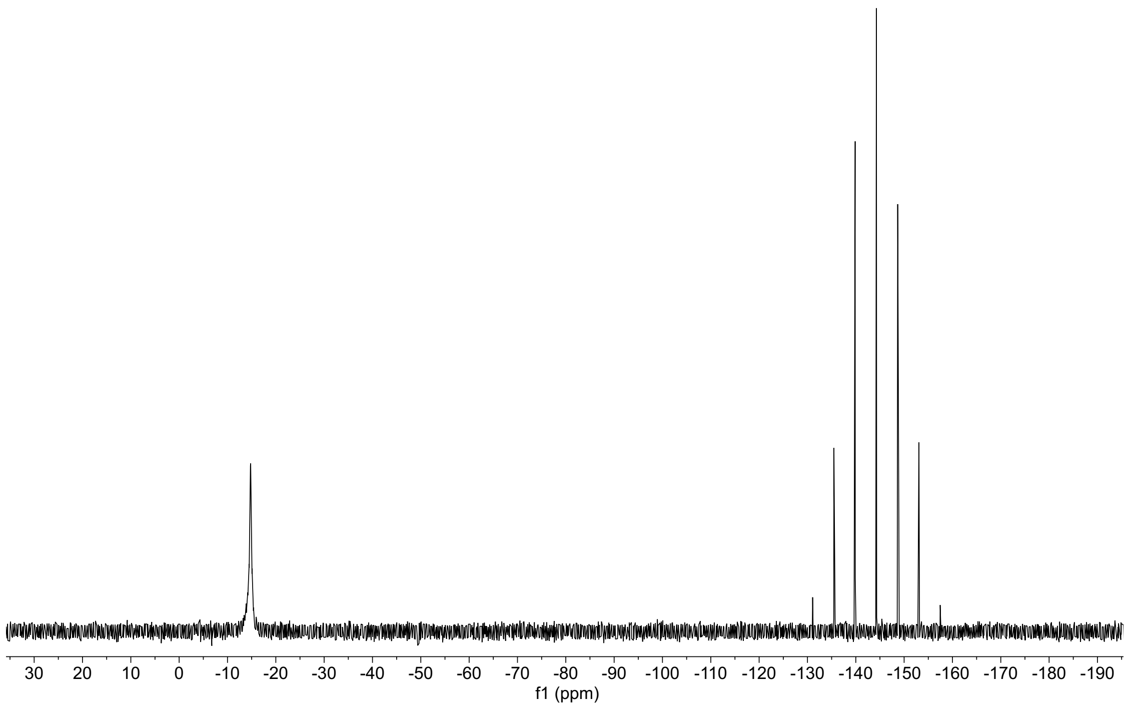

Figure S13. ^{31}P NMR (160 MHz, $CDCl_3$, 298 K)

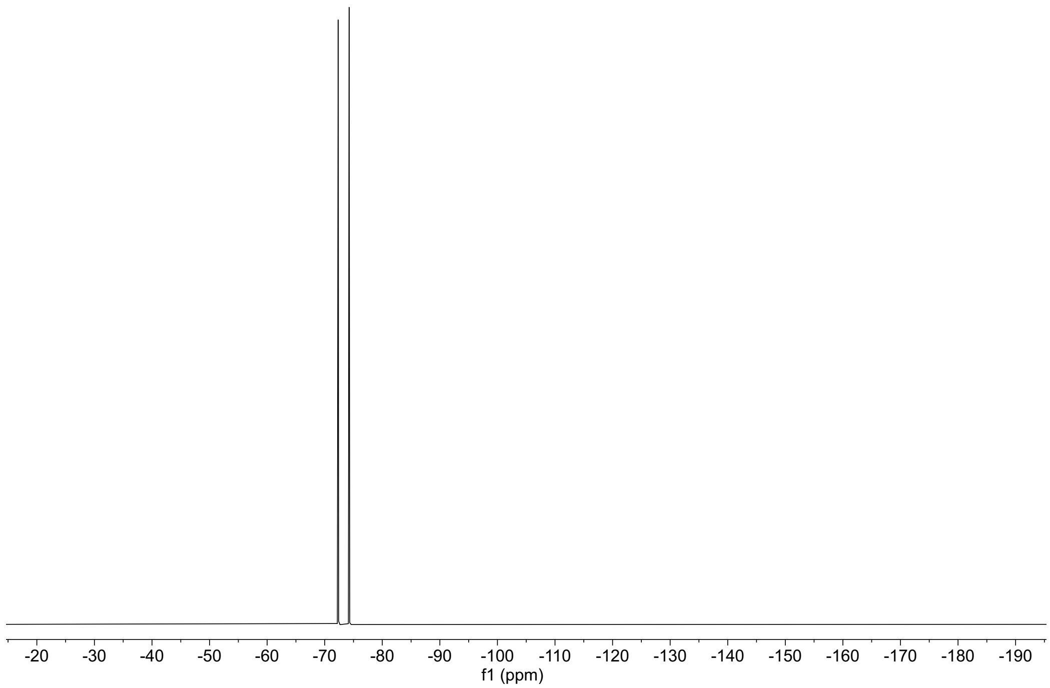

Figure S14. ^{19}F NMR (400 MHz, $CDCl_3$, 298 K)

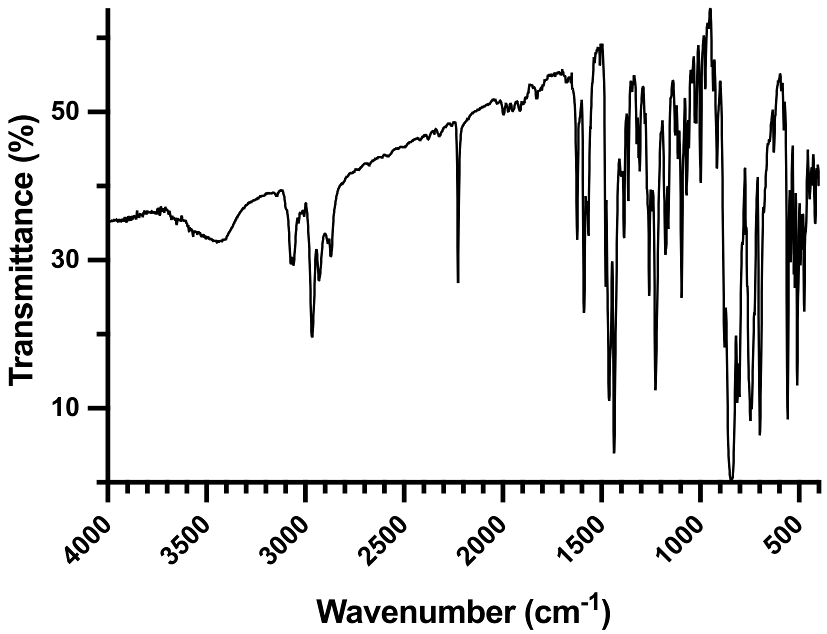

Figure S15. FT-IR spectrum of **CuC3** in KBr pellet.

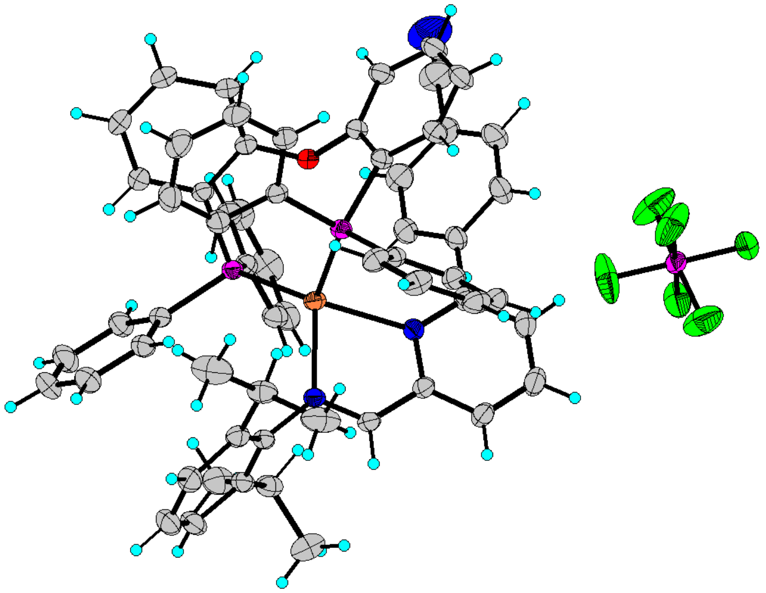

Figure S16. The asymmetric unit of **CuC3** with anisotropic ellipsoid representation. The ellipsoids are drawn at a 30% probability level, hydrogen atoms are depicted as spheres with arbitrary radii.

All the non-hydrogen atoms were refined with anisotropic displacement parameters. The hydrogen atoms were included from calculated positions and refined riding on their respective carbon atoms with isotropic displacement parameters. All geometrical calculations were done using the program Platon.[2] Besides, two disordered dichloromethane molecules were found in the asymmetrical unit and could not be satisfactorily refined. The SQUEEZE program from OLEX2 was therefore used to remove mathematically the effect of the solvent. The quoted formula and derived parameters do not include the squeezed solvent molecule.

Table S1. Crystal data, data collection, and structure refinement of the title compound.

	**CuC3**
CCDC number	2377087
Empirical formula	$C_{61}H_{53}CuN_3OP_2$, PF_6
Formula weight	1114.51
Temperature/K	296
Crystal system	monoclinic
Space group	$P2_1/n$ (No 14)
a/Å	21.4416(16)
b/Å	13.6023(10)
c/Å	22.4149(17)
β/°	111.671(10)
Volume/Å^3	6075.4(8)
Z'	1
Z	4
ρ_{calc}g/cm^3	1.219
μ/mm^{-1}	3.396
F(000)	2304
Crystal size/mm^3	0.20 × 0.25 × 0.30
Radiation	CuKα (λ = 1.54178)
2Θ range for data collection/°	4.1 to 72.6
Index ranges	-26≤ h ≤26, -16≤ k ≤16, -27≤ l ≤27
Reflections collected	178652
Independent reflections	11796 [R_{int} = 0.059]
Data/restraints/parameters	11796/0/681
Goodness-of-fit on F^2	1.05
Final R indexes [all data]	R_1 = 0.0614, wR_2 = 0.1867
Largest diff. peak/hole / e Å^{-3}	0.67/-0.47

Table S2. Bond distances (Å), angles (°), and torsion angles for the Cu(I) coordination sphere of compound **CuC3**.

Cu1-P1	2.3094(7)	Cu1-P2	2.2901(8)
Cu1-N2	2.270(2)	Cu1-N3	2.1073(18)
P1-Cu1-P2	114.17(2)	P1-Cu1-N2	98.82(6)
P1-Cu1-N3	118.56(6)	P2-Cu1-N2	128.14(6)
P2-Cu1-N3	114.13(6)	N2-Cu1-N3	78.85(7)
N2-Cu1-N3-C13	8.97(19)	N2-Cu1-N3-C14	166.8(2)

Emission spectra of complex CuC1 and NP.

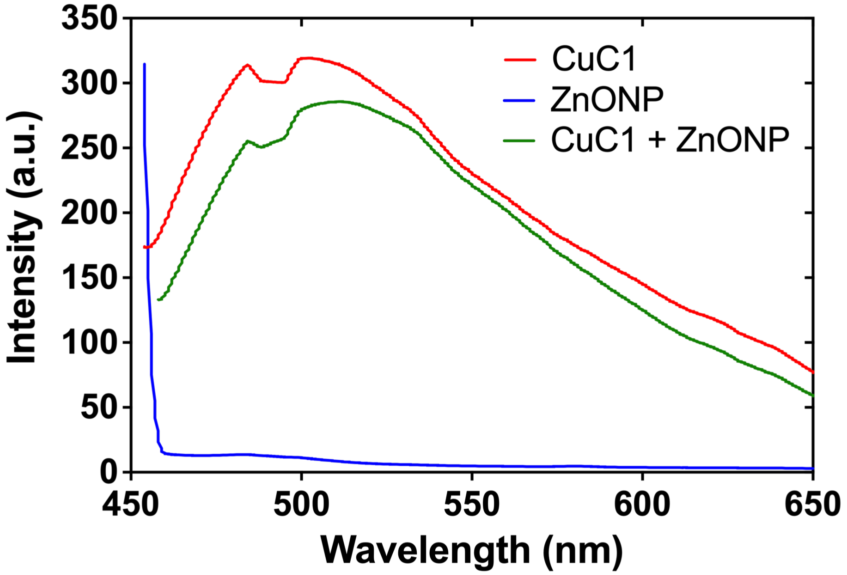

Figure S17. Emission spectra of CuC1, CuC1+ZnONP in acetone, and ZnONP in ethanol solution

References

[1] M. A. Escobar, D. Moreno da Costa, O. S. Trofymchuk, C. G. Daniliuc, F. Gracia, F. M. Nachtigall, L. S. Santos, R. S. Rojas, and A. R. Cabrera, Intermolecular stabilization in new 2-iminopyridine derivatives complexes of Pd(II) and their reactivity towards alkenes, J. Organomet. Chem., 863 (2018) 21-29. https://doi.org/10.1016/j.jorganchem.2018.03.032.

[2] L. Spek, Single-crystal structure validation with the program PLATON, J. Appl. Cryst. 36 (1) (2003) 7-13. https://doi.org/10.1107/S0021889802022112.
